# Genomic evaluations using data recorded on smallholder dairy farms in low- to middle-income countries

**DOI:** 10.3168/jdsc.2021-0092

**Published:** 2021-08-26

**Authors:** Owen Powell, Raphael Mrode, R. Chris Gaynor, Martin Johnsson, Gregor Gorjanc, John M. Hickey

**Affiliations:** 1 The Roslin Institute and Royal (Dick) School of Veterinary Studies, University of Edinburgh, Easter Bush, Midlothian, EH25 9RG, United Kingdom; 2 Scotland's Rural College (SRUC), Easter Bush, Midlothian, EH25 9RG, United Kingdom; 3 International Livestock Research Institute (ILRI), Nairobi 00100, Kenya; 4 Department of Animal Breeding and Genetics, Swedish University of Agricultural Sciences, Box 7023, 750 07, Uppsala, Sweden

## Abstract

Breeding has increased genetic gain for dairy cattle in advanced economies but has had limited success in improving dairy cattle in low- to middle-income countries (LMIC). Genetic evaluations are a central component of delivering genetic gain, because they separate the genetic and environmental effects of animals' phenotypes. Genetic evaluations have been successful in advanced economies because of large data sets and strong genetic connectedness, provided by the widespread use of artificial insemination (AI) and accurate recording of pedigree information. In smallholder dairy production systems of many LMICs, the limited use of AI and small herd sizes results in a data structure with insufficient genetic connectedness between herds to facilitate genetic evaluations based on pedigree. Genomic information keeps track of shared haplotypes rather than shared relatives captured by pedigree records. Therefore, genomic information could capture “hidden” genetic relationships, that are not captured by pedigree information, to strengthen genetic connectedness in LMIC smallholder dairy data sets. This study's objective was to use simulation to quantify the power of genomic information to enable genetic evaluation using LMIC smallholder dairy data sets. The results from this study show that (1) genetic evaluations using genomic information were more accurate than those using pedigree information in populations with a high effective population size and weak genetic connectedness; and (2) genetic evaluations modeling herd as a random effect had higher or equal accuracy than those modeling herd as a fixed effect. This demonstrates the potential of genomic information to be an enabling technology in LMIC smallholder dairy production systems by facilitating genetic evaluations with in situ records collected from herds of ≤4 cows. The establishment of routine genomic evaluations could allow the development of LMIC breeding programs comprising an informal set of nucleus animals distributed across many small herds within the target environment. These nucleus animals could be used for genetic evaluation, and the best animals could be disseminated to participating smallholder dairy farms. Together, this could increase the productivity, profitability, and sustainability of LMIC smallholder dairy production systems.

## Highlights

• Genomic evaluations outperformed pedigree-based genetic evaluations.
• Shared haplotypes captured "hidden" genetic relationships to strengthen connectedness in genomic evaluations.
• Genomic evaluations were possible using LMIC smallholder records from herds with ≤4 cows. . Modelling herd as a random effect produced EBVs with the highest accuracies.

## Graphical Abstract

**Figure:**
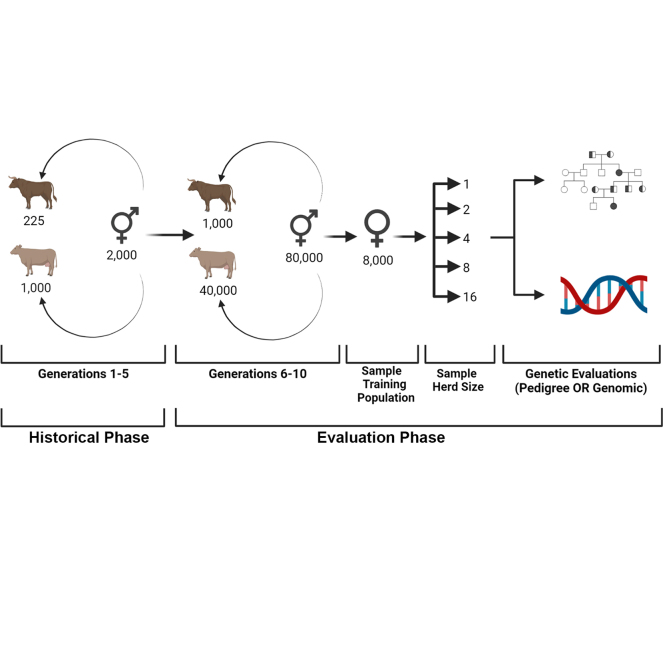
**Summary:** This study quantified the power of genomic information to enable genetic evaluation for smallholder dairy farmers in low and middle-income countries (LMICs). Stochastic simulations were used to generate pedigree, genotype, and trait information for populations with weak genetic connectedness and small herd sizes, reflecting the structure of LMIC dairy cattle datasets. Genomic and pedigree-based evaluations were compared on the accuracy of estimated breeding values for cows with own phenotypes.

The large increase in milk yield of dairy cattle in advanced economies over the past century is an example of the powerful effect that selective breeding can have on improving livestock productivity (Cole et al., 2020). For example, in the US dairy industry, milk production per cow approximately doubled between 1964 and 2004 (Ma et al., 2019). However, breeding practices have had poor efficacy and adoption in smallholder dairy production systems in many low- to middle-income countries (**LMIC**), despite the potential benefits. Recent estimates from Kenyan smallholder farms suggest that average milk production per cow is approximately 5 L/d, and there is little evidence of significant genetic improvement in recent decades (Kahi et al., 2004; Muriuki, 2011; Ojango et al., 2016). The low levels of productivity and its economic importance have renewed efforts to improve dairy cow productivity in LMIC smallholder dairy farms (East African Dairy Development Program, 2013; Rothschild and Plastow, 2014; Ducrocq et al., 2018).

Genetic evaluation is a central component of delivering genetic gain. The properties of an ideal data set that enables an accurate genetic evaluation include (1) genetic connectedness between herds or management groups (Kennedy and Trus, 1993); (2) large numbers of animals; and (3) large herd sizes. Such data enable the genetic and environmental effects of an individual animal's phenotype to be accurately separated. These features are not present in many LMIC smallholder dairy production systems. For example, smallholder dairy farmers in Kenya and other East African countries with ≤5 cows account for more than 70% of milk production (East African Dairy Development Program, 2013; Abdulsamad and Gereffi, 2016). Simultaneously, there is a low prevalence of AI use (5–10%; Ojango et al., 2014). Traditionally, this has prevented the establishment of effective pedigree-based genetic evaluation systems in these settings.

Genomic evaluations use a genomic relationship matrix to capture the realized, rather than expected, pedigree-derived relationships between animals (Nejati-Javaremi et al., 1997). The use of genomic information has enhanced many genetic evaluation systems in advanced economies. For example, the accuracy (the square root of reliability) of prediction for milk yield of young candidate bulls increased from 0.62 using pedigree-based BLUP (**PBLUP**) to 0.85 for genomic-based BLUP (**GBLUP**; Wiggans et al., 2017). In the context of LMIC smallholder dairy production systems, genomic data could be even more important than it has been in advanced economies. In such a setting, genomic data could capture and utilize information pertaining to haplotypes shared by animals in different herds. This information could reveal genetic connectedness that is unseen by pedigree information, which would, in turn, enable more accurate partitioning of the genetic and environmental effects on an animal's performance in small herds. Therefore, the use of genomic data could establish effective genetic evaluation systems based on data sets with relatively low levels of genetic connectedness (according to pedigree information).

Herd or management groups are usually included in the genetic evaluation model to enhance the separation of the genetic and systematic environmental effects of an animal's performance. Herds can be modeled as fixed or random effects (Schaeffer, 2009). Most genetic evaluations in advanced economies model herd as a fixed effect because herd sizes are typically large, which leads to fixed and random effect models giving almost equal solutions (Ugarte et al., 1992; Visscher and Goddard, 1993). When herd sizes are very small, such as in many LMIC smallholder dairy production systems, modeling herd as a fixed effect leads to an over-parameterized system of equations or inaccurate solutions (Oikawa and Sato, 1997). Modeling small herds as random effects may reduce this inaccuracy, yielding EBV with higher accuracies.

This study used simulation to quantify first the power of genomic information to enable genetic evaluation based on phenotypes recorded on smallholder dairy farms; and then, under such conditions, the impact of modeling herds as fixed or random effects. The simulations were performed using AlphaSimR (Gaynor et al., 2020) and were designed to (1) generate whole-genome sequence data, SNP, and QTL; (2) mate 1,000 sires per generation and vary the average herd size to generate pedigree structures with weak genetic connectedness to resemble LMIC smallholder dairy populations; and (3) run genetic evaluations modeling herd as either fixed or random effects. Ten independent replicates of the complete pipeline; that is, the simulation scheme and genetic evaluations, were completed. Code for the pipeline can be accessed at https://github.com/powellow/lmic_gblup. Conceptually, the simulation scheme was divided into historical and evaluation phases (Figure 1).

**Figure 1:**
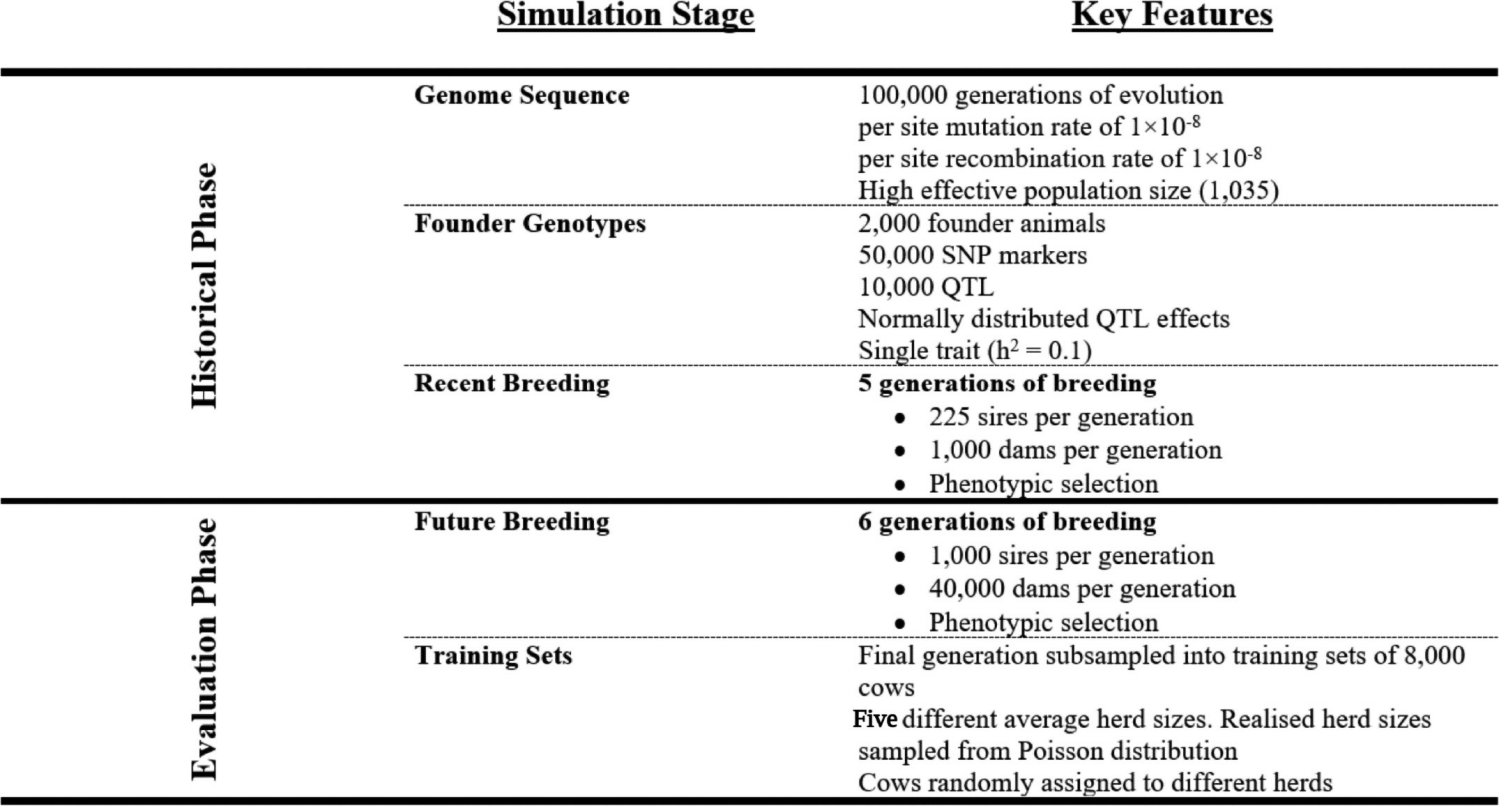
An overview of the simulation pipeline. Genome sequences were simulated for founder individuals and 10,000 segregating sites were assigned additive genetic effects for a low heritability trait representing total milk yield. Eleven generations of breeding were undertaken to generate a population with pedigree, genotype, and trait information. In the final generation, populations of 8,000 cows were assigned across herds of different sizes to generate the training sets for genetic evaluations..

A genome consisting of 10 chromosome pairs was simulated. The Markovian Coalescent Simulator (MaCS; Chen et al., 2009) and AlphaSimR were used to generate sequence data for 2,000 founder animals, with an effective population size (*N_e_*) of 1,035 in the final generation, to reflect the high genetic diversity found in cattle populations in Africa (Kim et al., 2017). The 2,000 founder animals served as the initial parents of the simulation. Segregating sites were randomly selected to serve as 5,000 SNP markers per chromosome (50,000 genome-wide in total) and 1,000 QTL per chromosome (10,000 genome-wide in total). A single record for the trait representing total milk yield from a single lactation was simulated for all animals. Therefore, no missing values were present in the data. The true breeding values (**TBV**) were calculated by summing the average effects of the animal's genotype at each QTL. The QTL additive effects were sampled from a standard normal distribution, N(0,1), and linearly scaled to produce TBV in the founder population with a variance $\left(\sigma_{a}^{2}\right)$ of 0.2. Herd and random error effects were sampled from normal distributions, resulting in a trait with a narrow-sense heritability of 0.1 and herd effect variance ratio of 0.4, chosen based upon previous literature (Ojango et al., 2019). The TBV, herd effects, and random error effects were summed to create the phenotypes of the animal.

Recent (burn-in) breeding for milk yield was simulated over 5 discrete generations of selective breeding on phenotype. The features of this breeding stage were 225 sires per generation, 1,000 dams per generation, and 2,000 offspring per generation. These numbers were chosen to match the base population (*N*_e_) of 1,035.

The evaluation phase of the simulation then modeled breeding with weak genetic connectedness for an additional 6 generations, following the common recent breeding burn-in phase. The common features across the evaluation phase were 1,000 sires mated per generation, offspring generated with an equal sex ratio, and, for simplicity, the selection of sires based on phenotype. Genetic connectedness was varied between different training populations by changing the average herd size. Herd sizes were sampled in 2 steps. In the first step, 8,000 samples were taken from a Poisson distribution with a lambda, the mean of the distribution, equal to 1, 2, 4, 8, and 16. However, stochasticity during the sampling process can result in the sum of the herd sizes differing from the size of training population of cows (8,000). Therefore, a second step randomly sampled herds to correct the difference between the sampled herd sizes and the required herd sizes to leave us with exactly 8,000 “slots” across all of the herds. This resulted in training populations with final average herd sizes of 1.58, 2.32, 4.06, 8, and 16. At the end of the simulation's evaluation phase, 8,000 phenotyped cows were randomly selected and randomly assigned to herds to serve as the training populations for the genetic evaluations. This number reflected the number of genotyped animals in the Africa Dairy Genetic Gains project at the time.

Breeding values were estimated using the following basic model:

[1] **y** = **Xb** + **Zu** + **e**,

where **y** is a vector of phenotype records measured on cows; **b** is a vector of fixed effects; **u** is a vector of breeding values for which we assumed that $u\text{˜}N\left(0,A\sigma_{a}^{2}\right)$ with PBLUP and $u\text{˜}N\left(0,G\sigma_{a}^{2}\right)$ with GBLUP, where **A** is the pedigree numerator relationship matrix (Henderson, 1975) based on the last 5 generations of error-free pedigrees, and **G** is the genomic numerator relationship matrix of individuals from the final generation of the evaluation phase (based on 50k SNP chip; VanRaden, 2008); **e** is a vector of residuals for which we assumed $e\text{\textasciitilde{}}N\left(0,I\sigma_{e}^{2}\right);$ **X** and **Z** are the incidence matrices linking phenotype records respectively to **b** and **u**; and **I** is the identity matrix. We adapted the basic model to create 3 genetic evaluation models in relation to a herd effect: (1) we excluded it, which gave us the basic model with intercept as the only fixed effect; (2) we modeled herd as a fixed effect; and (3) we modeled herd as a random effect, for which we assumed $h\text{\textasciitilde{}}N\left(0,I\sigma_{h}^{2}\right).$ All models included an overall intercept. We assumed that the variances of herd effects $\left(\sigma_{h}^{2}\right),$ breeding values $\left(\sigma_{a}^{2}\right),$ and residuals $\left(\sigma_{e}^{2}\right)$ were known, and we set them to the simulated values. Only phenotype data from generation 6 of the evaluation phase were used in genetic evaluations to mimic the recent introduction of phenotype, pedigree, and genomic data recording.

The PBLUP and GBLUP models were run using the Wombat software (Meyer, 2007). The different training populations and genetic evaluation models were compared based upon the accuracy of the EBV. We report mean and 95% interval of estimates over replicates. Accuracy was measured as the Pearson correlation coefficient between EBV and TBV.

The results from this study showed that genomic information enabled accurate genetic evaluation of phenotyped cows using data sets that comprised small herds with weak genetic connections. The main trends observed were that (1) genetic evaluations using genomic information had higher accuracy than those using pedigree information across all breeding designs; and (2) genetic evaluations with genomic information and modeling herd as a random effect had higher or equal accuracy compared with modeling herd as a fixed effect. The superiority of genetic evaluations using genomic information over pedigree information was consistent across trait heritabilities (h^2^ = 0.1, 0.3, and 0.5), but this superiority declined as heritability increased (data not shown). The superiority of modeling herd as a random effect was consistent across trait heritabilities (data not shown).

The genetic evaluation of phenotyped cows using genomic information had higher accuracy than that using pedigree information across breeding designs. Table 1 reports the accuracy of EBV of pedigree versus genomic evaluations as average herd size changed. The accuracies reported correspond to models in which herd was modeled as a random effect. At an average herd size of 1.58, phenotyped cows had an accuracy of EBV of 0.40 with PBLUP and 0.52 with GBLUP (an increase of 0.12). At all other average herd sizes, the increase in accuracy of GBLUP compared with PBLUP was between 0.12 and 0.13. Therefore, comparisons of different genetic evaluation models are only presented for GBLUP.

**Table 1:** The impact of genetic evaluation method on EBV accuracy^1^

Method	Size of herd				
	1.58	2.32	4	8	16
PBLUP	0.40	0.40	0.43	0.44	0.45
GBLUP	0.52	0.52	0.55	0.56	0.58

1 Comparison of the accuracy of genetic evaluation methods for training populations with different average herd sizes and using the pedigree (PBLUP) or genomic (GBLUP) method. Herd was modeled as a random effect. Standard errors were ≤0.01.

Genomic evaluations with herd modeled as a random effect had higher accuracy than modeling herd as a fixed effect at small average herd sizes. However, the accuracies of the 2 modeling approaches converged once a herd size of 8 was reached. Figure 2 plots the average herd size against accuracy for each of the 3 evaluation models. Figure 2 shows that excluding a herd effect gave an accuracy of 0.48, averaged across all herd sizes. At average herd sizes of 1.58 and 2.32, modeling herd as a random effect increased the accuracy by 0.10 and 0.05, respectively, compared with modeling herd as a fixed effect. At an average herd size of 8, the accuracies from the 2 modeling approaches practically converged.

**Figure 2:**
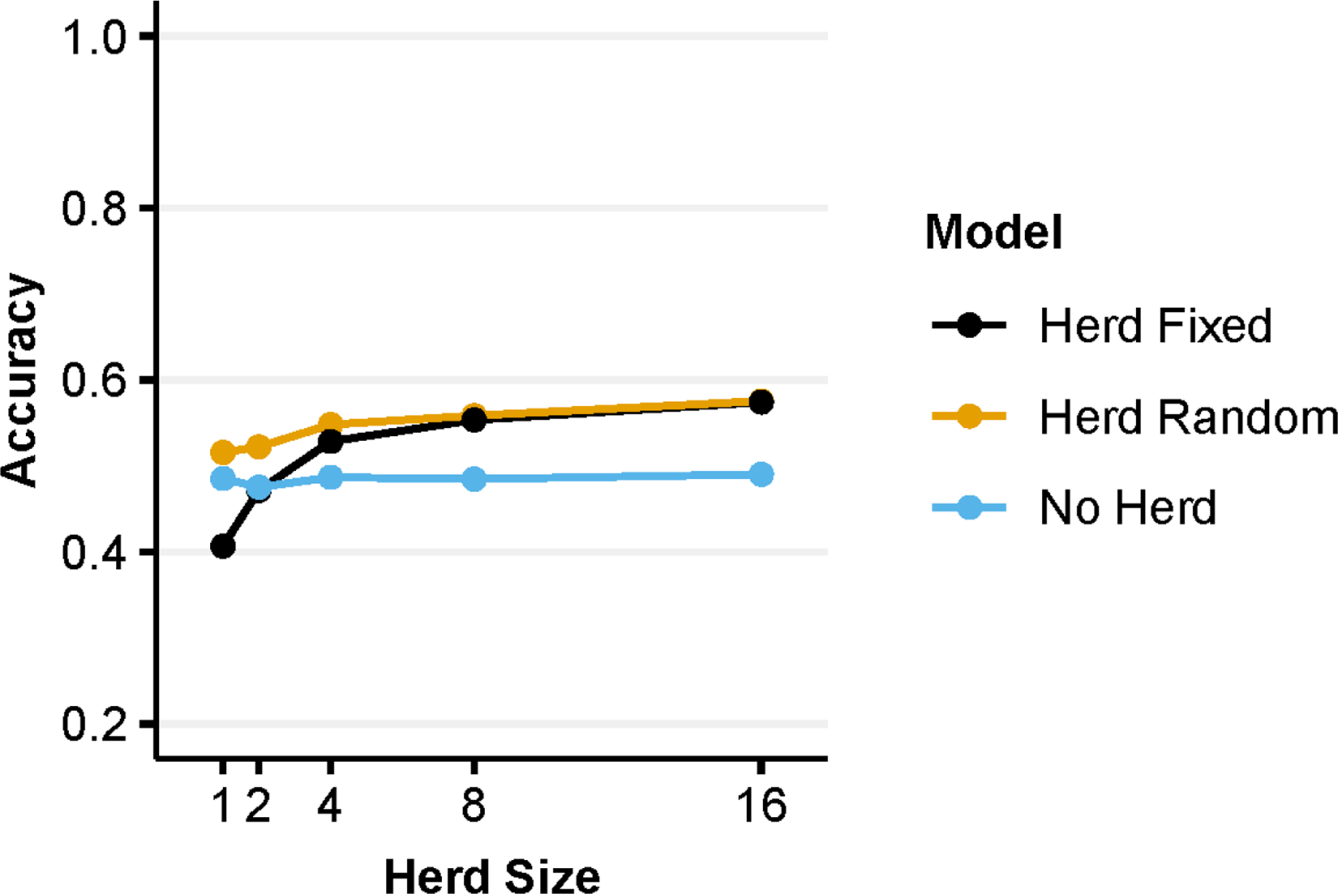
A comparison of the statistical modeling of herd effects with genomic BLUP (GBLUP), showing the accuracy of EBVs for training populations with different average herd size (1–16) when the herd effect was (1) excluded from the model (No Herd), (2) modeled as a fixed effect (Herd Fixed), and (3) modeled as a random effect (Herd Random).

Our results demonstrate that genomic evaluations could be effective for dairy cattle populations with weak genetic connectedness, small herd sizes, and low heritability traits. The improvement in the accuracies of EBV with genomic evaluations (Wiggans et al., 2017) compared with pedigree-based evaluations and modeling small herds as random effects have separately been shown previously in advanced economies (Ugarte et al., 1992; Visscher and Goddard, 1993; Schaeffer, 2009). We have confirmed these previous findings and extend them by demonstrating that genomic evaluations provide accurate EBV when using data structures representative of LMIC smallholder dairy production systems. Such smallholder dairy production systems will further benefit from using genomic evaluations compared with pedigree-based evaluations because of implicit increases in genetic connectedness between very small herds as a result of tracking shared haplotypes rather than shared relatives. The increases in genetic connectedness result in lower confounding between genetic and nongenetic effect estimates. However, our simulations did not model the full complexity of practical genetic evaluations for LMIC smallholder dairy production systems. A limitation of the current study is the assumption of the quality of phenotypes. We partially reflected the low quality of phenotypes by simulating a trait heritability of 0.1, based on empirical data (Ojango et al., 2019). However, we ignored the impact of missing data. Therefore, this study's results, dependent on the accurate estimation of variance components, should be validated with empirical data. Projects such as Africa Dairy Genetic Gains, with ongoing data collection efforts, are in a prime position to do so.

The establishment of effective genomic evaluations could enable in situ data recorded on smallholder farms to be used to drive in situ genetic improvement for LMIC target production environments. Such LMIC breeding programs could comprise an informal set of nucleus animals distributed across a subset of small herds within the target environment. These nucleus animals could be used for the genetic evaluation and the best animals disseminated to participating smallholder dairy farms. Together, this would increase the productivity, profitability, and sustainability of LMIC smallholder dairy production systems.

However, the infrastructure required for such breeding programs and the associated technologies is expensive, potentially creating a new cost barrier to animal breeding success in LMIC smallholder dairy production systems. New business models are needed to overcome this barrier in a self-sustaining way. These business models could bundle technology, data recording, extension services, and a marketplace for LMIC smallholder farmers. This type of self-sustaining platform would maximize the benefits and cost-efficiency of any component (e.g., the genotyping and phenotyping of animals). The Africa Dairy Genetic Gains and Public Private Partnership for AI Dissemination (PAID, 2017) projects, emerging social enterprises (e.g., One Acre Fund; https://oneacrefund.org/), and electronic marketplaces for agricultural products in LMICs (e.g., Livestock 247; https://livestock247.com) show that many components of such a model are already in place.
